# Advancements and Innovations in the Surgical Management of Sacrococcygeal Pilonidal Sinus: A Comprehensive Review

**DOI:** 10.7759/cureus.61141

**Published:** 2024-05-26

**Authors:** Simran Dhole, Chanrashekhar Mahakalkar

**Affiliations:** 1 General Surgery, Jawaharlal Nehru Medical College, Datta Meghe Institute of Higher Education and Research, Wardha, IND

**Keywords:** minimally invasive, recurrence, innovations, advancements, surgical management, sacrococcygeal pilonidal sinus

## Abstract

Sacrococcygeal pilonidal sinus (SPS) is a common condition characterized by the formation of a sinus tract or cavity in the sacrococcygeal region, often containing hair and debris. Surgical management plays a crucial role in its treatment due to its chronic and recurrent nature. This comprehensive review explores the advancements and innovations in the surgical management of SPS. The review begins with an overview of the historical perspective, anatomy, and pathophysiology of the condition, followed by a discussion of current surgical techniques, including conventional excision, flap procedures, and minimally invasive approaches. Recent advancements, such as laser therapy, radiological guidance techniques, and robotic-assisted surgery, are also examined. The key findings from outcomes research are summarized, including postoperative pain management, recurrence rates, and patient satisfaction. The implications for clinical practice are discussed, emphasizing the importance of staying updated on the latest surgical techniques and adopting a personalized approach to treatment. Recommendations for future research are provided, highlighting the need for prospective studies comparing different surgical techniques, as well as research focusing on minimally invasive approaches and predictive models for recurrence risk. Collaboration among researchers, clinicians, and industry partners is essential to drive innovation and improve outcomes for patients with SPS.

## Introduction and background

The sacrococcygeal pilonidal sinus (SPS) is a common condition characterized by the formation of a sinus tract or cavity in the sacrococcygeal region, typically containing hair and debris. This condition often presents as a chronic inflammatory process and can lead to recurrent abscesses and sinus tract formation [1]. The prevalence of SPS varies widely across different populations, with estimates ranging from 26 to 181 cases per 100,000 individuals. Incidence rates are typically highest among young adults, particularly males, and may be influenced by factors such as obesity, sedentary lifestyle, and genetic predisposition [2].

Surgical management plays a crucial role in the treatment of SPS due to the chronic and recurrent nature of the condition. While conservative measures such as antibiotics and local wound care may provide temporary relief, surgical intervention is often necessary to achieve long-term resolution and prevent complications such as abscess formation, chronic pain, and impaired quality of life [3]. The purpose of this review is to provide a comprehensive overview of the advancements and innovations in the surgical management of SPS. By examining the evolution of surgical techniques, current treatment modalities, outcomes, and emerging technologies, this review aims to elucidate the state-of-the-art approaches in the field and identify areas for future research and development.

## Review

Historical perspective

Early Surgical Techniques

The historical progression of surgical techniques for SPS disease has witnessed a series of approaches aimed at enhancing outcomes and diminishing recurrence rates. Initially, surgical interventions comprised incision and drainage, excision of afflicted tissue with primary closure, and excision with secondary healing [4]. Subsequent advancements prompted comparisons among various methods, such as marsupialization, the lay-open technique, vertical excision with primary closure, Limberg flap transposition, and Karydakis flap transposition [5]. A notable meta-analysis spanning over 180 years of data on pilonidal sinus treatment underscored the correlation between recurrence rates and follow-up durations for diverse surgical procedures, highlighting the significance of prolonged monitoring and the variability in recurrence rates among different techniques [5]. The historical examination of early surgical techniques for SPS underscores the ongoing pursuit to pinpoint the most efficacious approach for managing this condition, with a shift towards off-midline flap reconstructions and innovative modalities like laser treatments and sclerosing agents. This journey reflects the relentless endeavors to optimize patient outcomes and alleviate the burden of SPS through evidence-based surgical interventions [4].

Evolution of Surgical Approaches

The evolution of surgical approaches for SPS treatment has been characterized by a persistent pursuit of identifying the most effective and least invasive methods. Throughout the years, a plethora of surgical techniques have emerged and undergone comparison to tackle the challenges inherent in SPS management. While once prevalent, the conventional midline closure technique has demonstrated a heightened incidence of postoperative recurrence, prompting a transition towards more sophisticated and customized approaches [4]. Recent research endeavors have extensively analyzed and contrasted various surgical methods, including primary closure, Limberg flap, Karydakis flap, and others, elucidating each approach's advantages and drawbacks concerning recurrence rates, duration of return to work, and overall treatment outcomes [6]. For instance, primary closure has exhibited elevated recurrence rates compared to techniques like Karydakis and Limberg flaps, which have demonstrated lower recurrence rates and shorter return periods [6]. In optimizing the surgical management of SPS, personalized treatment plans have become indispensable, incorporating considerations such as the recurrence rates associated with different closure techniques and the necessity for tailored approaches based on patient-specific conditions and the surgeon's proficiency [4]. The ongoing evolution in surgical approaches for SPS epitomizes a continual endeavor to enhance patient outcomes, diminish recurrence rates, and elevate the overall efficacy of treatment.

Anatomy and pathophysiology

Anatomy of the Sacrococcygeal Region

The sacrococcygeal region encompasses the sacrum and coccyx, with the sacrococcygeal joint serving as a pivotal articulation between the sacrum's apex and the coccyx's base [7,8]. This joint, classified as a symphysis, is characterized by hyaline cartilage lining the bones and an interposed fibrous disc, facilitating limited movement primarily for flexion and extension [7]. Significantly, the sacrococcygeal joint increases the anteroposterior diameter of the pelvis during labor and defecation [7]. Anatomically, its integrity is reinforced by ligaments such as the anterior sacrococcygeal, superior posterior sacrococcygeal, deep posterior sacrococcygeal, lateral sacrococcygeal, and intercornual ligaments [7]. Innervated by spinal nerves S4-Co and supplied by the inferior lateral sacral and median sacral arteries, this joint plays a critical role in pelvic stability and function [7]. The coccyx at the spine's base comprises fused coccygeal vertebrae and measures approximately 4-10 cm long, forming an inverted triangle shape with the base on top and the apex below [9]. Functionally, the coccyx is involved in movements such as shifting forward during sitting to provide shock absorption and moving backward during childbirth to facilitate delivery [9]. However, coccydynia, or tailbone pain, can sometimes arise due to unstable coccyx, sacrococcygeal joint inflammation, or bony spurs' growth on the coccyx [9].

Pathogenesis of Pilonidal Sinus

The pathogenesis of pilonidal sinus entails the infection or inflammation of hair follicles located within the intergluteal cleft, culminating in creating a sinus connected to the skin surface via an epithelialized sinus tract [10,11]. This condition predominantly affects young adults, particularly males in their 20s, and is more prevalent among individuals with coarse, dark body hair who engage in prolonged periods of sitting [10]. Clinically, the disease manifests as a discharging sinus in the sacrococcygeal region, often accompanied by intermittent pain and the secretion of serous fluid, which may evolve into bloody or purulent discharge [10]. The pathogenesis begins with the obstruction of a hair follicle in the intergluteal cleft, leading to the formation of a pit that extends inward, eventually giving rise to a cavity connected to the skin surface by a sinus tract [10]. Contributing factors to the development of pilonidal sinus disease (PSD) include increased sweating, friction in the buttock area, obesity, and poor hygiene practices [12]. This pathogenic process is characterized by a foreign body-type reaction, which can precipitate the formation of cysts that may undergo acute infection, resulting in pilonidal abscesses [10].

Factors Contributing to Disease Development

**Figure 1:**
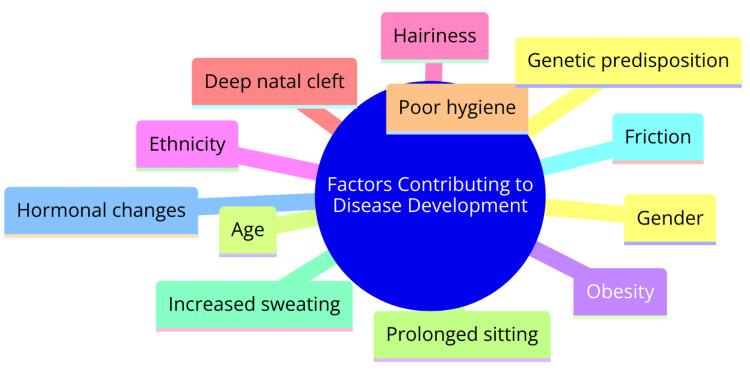
Factors contributing to disease development Image credit: Dr Simran Dhole

SPS disease development involves a complex interplay of risk factors and pathophysiological mechanisms. Risk factors contributing to the condition encompass male gender, young age, obesity, Mediterranean ethnicity, hairiness, deep natal cleft, and poor hygiene practices [3]. Predominantly affecting males, the disease typically manifests during adolescence, with approximately 1% of all males and 0.1% of all females harboring an asymptomatic pilonidal sinus with the potential for disease development [13]. Pathophysiologically, the condition entails the chronic infection of hair follicles in the sacrococcygeal region. Repeated movements of the buttocks cause hair to penetrate follicles, instigating the formation of a foreign body granuloma and subsequent infection [3]. The shape of the natal cleft and buttock movements facilitates the embedding of barbed hairs into sinuses, exacerbating the infection, and acting as foreign bodies [13]. While the precise etiology of PSD remains incompletely understood, it is believed to be associated with hormonal changes leading to the enlargement of hair follicles and subsequent blockage in the pilosebaceous glands within the sacrococcygeal region [13]. The development of SPS disease is influenced by a myriad of factors, including anatomical considerations, individual characteristics, and underlying pathophysiological processes, all of which contribute to the initiation and progression of this condition. Factors contributing to disease development are shown in Figure 1.

Current surgical techniques

Conventional Excision and Closure

Conventional excision and closure represent a widely employed surgical technique for managing diverse skin conditions, encompassing PSD and cutaneous tumors. This method entails the excision of diseased tissue followed by primary wound closure, which can be achieved through various approaches, such as triangular excision with primary closure [14]. Compared to alternative surgical modalities, such as flap and graft techniques, conventional excision and closure are often lauded for their efficacy in treating cutaneous tumors while preserving adjacent skin and facilitating the adaptation of suture lines in areas with heightened tension [14]. In PSD treatment, conventional excision and closure constitute one of the traditional therapeutic options, encompassing excision with midline closure, off-midline closure employing various subcutaneous flap techniques, and excision with secondary intention healing [15]. Nevertheless, PSD is notorious for its propensity for recurrence post-excision, necessitating recurrent procedures, and prolonged periods of sick leave [15]. The emergence of minimally invasive approaches, such as laser ablation, has garnered increased attention in recent years, offering advantages such as reduced trauma and expedited recovery times following the procedure [15]. A comparative study evaluating laser ablation against simple excision with direct closure, and excision with flap reconstruction in PSD treatment, revealed that laser ablation exhibited a lower complication rate than the other two methods [15]. However, the laser group demonstrated a significantly higher incidence of residual disease. This study underscores the benefits of laser treatment, including swift postoperative recovery and diminished risk of complications [15]. While conventional excision and closure serve as a longstanding surgical approach for PSD, they are not devoid of limitations, notably the risk of disease recurrence. Adopting minimally invasive techniques, like laser ablation, presents promising outcomes characterized by fewer complications and accelerated recovery periods [15].

Limberg Flap Procedure

The Limberg flap procedure is a surgical technique that addresses conditions like SPS disease. This method involves crafting a flap equivalent in size to the defect slated for excision, with all angles set at 60°, thereby mitigating tension and averting complications such as tension blisters [16]. Characterized by a design resembling a series of interconnected equilateral triangles, the Limberg flap enables the closure of rhomboid defects with minimal tension, culminating in optimal aesthetic outcomes [16]. Empirical evidence has underscored the efficacy of the Limberg flap in reconstructing defects after the excision of SPS, yielding low complication rates and favorable patient outcomes [17]. Comparative analyses have juxtaposed the Limberg flap procedure against alternative techniques like the Karydakis flap, yielding disparate recurrence rates and durations of hospital discharge across various studies [18]. The Limberg flap procedure emerges as a secure and dependable option for the surgical management of SPS disease, offering advantages such as diminished recurrence rates and mitigated postoperative complications [18].

Bascom's Pit Picking Technique

Bascom's Pit Picking Technique, or Bascom I, represents a minimally invasive surgical strategy employed in treating PSD. This approach entails the meticulous excision of all pits down to the underlying cavity through small individual incisions to preserve as much normal skin as feasible. Central to the procedure is thoroughly cleansing the cavity of hairs and granulation tissue, leaving the resulting wounds open for secondary intention healing [19]. Bascom's adaptation of this technique incorporates the creation of an additional incision lateral to the midline, facilitating the removal of inflamed tissue from the cavity and its capsule. Following this, the wounds along the midline are sutured closed, while the lateral incision is deliberately left open to facilitate drainage [19]. Studies have reported 24% to 29% recurrence rates with this method [19]. Bascom's Pit Picking Technique has demonstrated favorable outcomes among patients with uncomplicated PSD, boasting a high rate of treatment success and low incidences of postoperative complications and chronic pain [20]. This technique is deemed a suitable treatment option for most patients presenting with a simple pilonidal sinus, underscoring its efficacy in managing this condition [20].

Karydakis Flap Procedure

The Karydakis flap procedure is a surgical cornerstone in managing SPS disease. This technique entails an asymmetric excision of the affected region, followed by the off-midline closure of the wound using a lipocutaneous flap to flatten the natal cleft, primarily mitigating recurrence rates associated with SPS disease [21,22]. Since its inception in 1968, the Karydakis flap has undergone refinement, now encompassing two-layered fat closure, meticulous thin flap creation, displacement of the caudal apex, avoidance of routine drainage, and utilization of absorbable skin sutures [21]. Evidentiary support underscores the efficacy of the Karydakis flap in achieving a low recurrence rate by fostering scar formation away from the midline, rendering it applicable for all types of pilonidal disease [21]. However, notwithstanding its effectiveness, the Karydakis flap procedure poses challenges such as wound dehiscence and the technical intricacy of the procedure, underscoring the imperative for surgical proficiency and meticulous patient selection [22]. Adaptations and modifications of the Karydakis flap have been explored to address specific patient scenarios, particularly secondary sinuses situated distally from the midline, where alternative procedures such as rotational flaps may offer more excellent suitability [22]. The Karydakis flap procedure remains an invaluable component of the surgical armamentarium for SPSD management, proffering reproducibly low recurrence rates and favorable outcomes when executed with precision and scrupulous attention to detail [22].

Endoscopic and Minimally Invasive Approaches

Endoscopic and minimally invasive approaches have emerged as promising alternatives in managing SPS, offering potential benefits such as reduced postoperative pain, shortened recovery times, and improved cosmetic outcomes, compared to traditional open surgical methods [23-25]. One such technique is the endoscopic pilonidal sinus treatment (EPSiT), which involves the insertion of a fistuloscope into the sinus tract to identify and remove hair, followed by cauterization of the tract [23]. Studies have reported favorable short-term outcomes with EPSiT, including minimal postoperative pain, favorable aesthetic results, low infection rates, and high patient satisfaction [23]. Another minimally invasive approach, video-assisted ablation of the pilonidal sinus (VAAPS), employs a fistuloscope to visualize and treat the sinus tract. A case report demonstrated successful treatment of a patient with VAAPS, resulting in good healing and no recurrence at a 10-month follow-up [23]. While these minimally invasive techniques offer promising results, long-term data on recurrence rates are still needed to establish their efficacy compared to traditional surgical methods [23]. Furthermore, the availability of specialized equipment and the associated learning curve may limit their widespread adoption [23]. Endoscopic and minimally invasive approaches for SPS treatment exhibit encouraging short-term outcomes, but further research is necessary to determine their long-term efficacy and optimal patient selection criteria. These techniques should be considered part of the surgical armamentarium for SPS, particularly in cases of limited disease [23].

Advancements in surgical management

Laser Therapy

Laser therapy has emerged as a promising minimally invasive treatment modality for SPS. A systematic review and meta-analysis highlighted the efficacy of laser ablation utilizing a 1470 nm radial diode laser fiber, demonstrating a healing rate of 100% with an average healing time of 28.3 ± 5.5 days [26]. This procedure involves creating a small incision and using the laser fiber to seal the sinus tract [27]. Studies have reported impressive complete healing rates of up to 94.4% with laser treatment alone [28]. The exploration of combined laser therapy with phenol application has also shown promise. In a retrospective study, the laser-phenol treatment group exhibited a lower recurrence rate (4.8%) than laser treatment alone (13.6%), albeit without statistical significance. Additionally, the laser-phenol group demonstrated a higher complete healing rate (95.5%) in contrast to the laser-only group (91.7%) [28]. Laser therapy presents several advantages, including minor wounds, mild postoperative discomfort, and a short recovery period, enabling patients often to resume work within a week post-surgery [27]. Nevertheless, concerns regarding recurrence rates persist, necessitating further investigation to ascertain laser therapy's position as the gold standard for SPS treatment [26,28].

Radiological Guidance Techniques

Radiological guidance techniques are pivotal in managing SPS disease. Magnetic resonance imaging (MRI), particularly utilizing T2-weighted images, is a valuable tool in assessing and diagnosing SPS, aiding in its differentiation from other conditions such as fistula in ano [29]. This imaging modality offers crucial insights into the disease's extent, facilitating treatment planning and informed decision-making. Furthermore, radiological guidance techniques are indispensable in surgical interventions for SPS, notably in procedures like the lateral advancement flap technique. This approach entails the advancement of a fascia-adipose-cutaneous flap across the natal cleft to achieve off-midline closure. MRI imaging assumes a vital role in both preoperative assessment and postoperative evaluation of outcomes, contributing to the precision and efficacy of the procedure [30]. Incorporating radiological guidance techniques, such as MRI imaging, significantly enhances the accuracy and effectiveness of surgical interventions for SPS. Consequently, this leads to improved patient outcomes and reduced recurrence rates, underscoring the indispensable role of such techniques in the management of SPS.

Bioengineered Tissue Grafts

Bioengineered tissue grafts, referred to as bioengineered skin grafts or substitutes, mark a significant stride in regenerative medicine. These grafts represent a paradigm shift in therapeutic approaches, specifically tailored to address the urgent need for effective wound healing and tissue regeneration, especially in deep wounds where conventional treatments may fall short [31-33]. The quintessential bioengineered skin substitute for deep wounds is envisioned to comprise three layers mirroring the anatomical and functional characteristics of native skin. This includes a hypodermal-like layer to bolster vascularization and foster expeditious wound healing [31]. Comprising diverse materials such as human tissue (autologous or allogeneic), non-human tissue (xenographic), synthetic materials, or a composite thereof, these grafts are embedded within a cellular or acellular matrix to provide structural support [31]. As temporary or permanent wound coverings, bioengineered skin grafts catalyze new skin growth and expedite wound healing by introducing living cells that reinstate a moist wound environment and furnish structural reinforcement [31]. The evolution of these grafts endeavors to furnish personalized and efficacious therapies that elevate patient outcomes and enhance quality of life, with ongoing endeavors aimed at standardizing fabrication processes and curbing manufacturing costs [31,33]. Representing a promising frontier in tissue engineering, bioengineered skin grafts serve as a bridge between conventional skin grafting techniques and advanced regenerative medicine modalities, proficiently addressing the complexities associated with wound healing challenges.

Robotic-Assisted Surgery

Advancements in robotic-assisted surgery have ushered in a transformative era in surgical management. The FDA approval of the da Vinci Surgical System in 2000 marked a watershed moment, empowering surgeons to execute intricate procedures with heightened precision and control [34]. Leveraging robotic systems like the da Vinci system, robot-assisted surgery spans a spectrum of procedures across diverse medical specialties, encompassing general, abdominal, cardiac, urological, gynecological, and otolaryngological surgeries [35]. The amalgamation of artificial intelligence (AI) into surgical technology is revolutionizing the capabilities of human surgeons, yielding benefits such as diminished technical errors, augmented outcomes, and augmented access to challenging anatomical regions [36]. The future trajectory of robotic surgery is promising, characterized by ongoing advancements in miniaturization, enhanced imaging modalities, intelligent systems, and less invasive methodologies. These developments culminate in more precise and efficient surgical interventions [37]. The exploration of 5G networks' potential in robotics is underway, envisaging near-instantaneous communication capabilities that could facilitate remote procedures, elevate quality standards, and expand healthcare accessibility [37]. The synergy of professionals from diverse domains, including healthcare, engineering, and technology, is paramount in propelling the frontier of robot-assisted surgery and realizing life-altering outcomes [37].

Novel Wound Closure Methods

Laser-tissue welding is a burgeoning technique that achieves meticulous wound closure by concurrently coagulating and cauterizing the tissue [38]. This method holds significant promise and offers advantages such as rapid closure, minimal risk of foreign body reaction, and ease of use even in emergencies. Studies have indicated that a pulsed 980 nm laser yields lesser thermal damage and superior closure outcomes compared to 1064 nm lasers [38]. Skin flaps represent a standard modality for wound closure, encompassing various types such as advancement, rotation, island pedicle, and transposition flaps [39]. These techniques involve mobilizing tissue from adjacent regions to cover the wound, enhancing function by managing skin tension, and improving cosmesis by concealing scars. Alternatively, skin grafts provide another avenue, where the skin is excised from one site and transplanted into the wound defect [39]. Full-thickness grafts incorporate both epidermis and full dermis, while split-thickness grafts are thinner. Successful grafting necessitates a well-vascularized wound bed. Suture techniques continue to evolve, with innovative approaches aimed at distributing tension along the wound edge, especially in high-tension wounds or delicate skin [40]. A commonly employed method involves burying dissolving sutures in the dermis or deeper layers, followed by dissolving or non-dissolving sutures under minimal tension to oppose the epidermis [39]. Wound closure typically entails a two-layer process [1]. The initial layer employs absorbable sutures in the deeper tissues, while the subsequent layer utilizes either absorbable or non-absorbable sutures to align the skin edges precisely [39]. Adhesive tapes and skin glues can complement closure techniques to reinforce the integrity of the closure [39].

Outcomes and complications

Postoperative Pain Management

Postoperative pain management stands as a pivotal facet of patient care in the aftermath of surgery. It entails a multifaceted approach involving diverse medications and techniques aimed at mitigating pain and fostering a seamless recovery process. Anesthesiologists assume a central role in this endeavor, administering pain-relieving medications intravenously (IV), typically commencing with opioids to manage initial pain levels. Tailored pain medication regimens, whether in pill form or otherwise, are orchestrated based on factors such as the nature of the surgery, pain intensity, patient health status, and medical history [41]. Beyond opioids, a spectrum of prescription and over-the-counter medications, including ibuprofen, acetaminophen, and aspirin, are commonly deployed to alleviate postoperative pain. These medications not only assuage pain but also address inflammation and swelling, thus improving pain relief [41,42]. Complementary non-medication interventions, such as heating pads, ice packs, and relaxation techniques, are also integrated into pain management strategies in conjunction with or as alternatives to pharmacological approaches [41]. Multimodal analgesic techniques, blending diverse pain relief modalities, are frequently advocated for postoperative pain management. Regional anesthetic methodologies, such as epidural analgesia and perineural techniques, have emerged as efficacious means to address postoperative pain, with infiltrative techniques demonstrating utility across a spectrum of surgical procedures [43]. Customizing postoperative pain management plans for individual patients is paramount, factoring in the specific surgical procedure, pain characteristics, and patient-specific requirements. Adequate pain control fosters comfort, expedites recovery, mitigates the risk of complications, and enhances overall patient satisfaction and outcomes [44].

Recurrence Rates

Recurrence rates following diverse surgical interventions for PSD have been subject to extensive scrutiny. A comprehensive analysis involving over 80,000 patients underscored the necessity of diligent follow-up to ascertain precise recurrence rates after specific procedures [3]. Notably, the study unveiled that advancement flap procedures boasted the lowest recurrence rates across all follow-up durations, ranging from 0.2% at 12 months to 1.9% at 60 months. In stark contrast, excision with primary midline closure exhibited the highest long-term recurrence rates, escalating to 67.9% at 240 months, prompting recommendations to prioritize off-midline closure or flap techniques over midline closure [3]. Geographical variances have emerged as influential determinants of recurrence rates in PSD, with primary asymmetric closure and an array of flap techniques manifesting superior outcomes across distinct regions [45]. The global dispersion of surgical methodologies assumes significance in recurrence rates, with discernible discrepancies observed among nations and surgical modalities [45]. For instance, in the USA, recurrence rates ranged from 0.3% for Karydakis/Bascom approaches to 67.2% for incision procedures [45]. The selection of surgical approach, duration of follow-up, and geographic locale constitute pivotal determinants shaping recurrence rates in PSD. Tailored treatment strategies, cognizant of these variables, stand poised to curtail recurrence and enhance patient outcomes.

Wound Healing and Complication Rates

The comparative study contrasting the Karydakis flap technique with partial primary closure (PPC) modified with a suture technique for SPS treatment unveiled analogous total complication rates between the two methodologies, registering 30.4% in PPC and 30% in Karydakis flap. Nevertheless, the PPC technique exhibited a protracted healing duration, averaging 36.67 ± 8.09 days [46]. Concurrently, a separate investigation concerning sacrococcygeal hygiene post-surgery underscored the pivotal role of meticulous wound hygiene in facilitating optimal healing devoid of complications. The study advocated postoperative water irrigations in the intergluteal cleft to curtail healing duration and complication incidences, accentuating the indispensable significance of sacrococcygeal hygiene in PSD management [47]. While the comparative analysis between PPC and Karydakis flap techniques revealed akin complication rates, it delineated a prolonged healing period with PPC. Conversely, exploring sacrococcygeal hygiene emphasized the criticality of robust wound hygiene practices in fostering favorable postoperative healing outcomes.

Future directions and emerging technologies

Gene Therapy and Immunomodulation

Gene therapy and immunomodulation represent interconnected domains holding immense potential for treating diverse diseases. Immunomodulation is pivotal in gene therapy, particularly in orchestrating immune responses to the viral vectors employed in gene delivery. The intricate interplay between the immune system and gene therapy vectors is a linchpin for the efficacy and safety of gene therapy interventions. Strategies encompassing immunosuppression, immune tolerance induction, and antigen-selective modulation have been meticulously explored to bolster gene therapy's efficacy and safety profiles, notably with adeno-associated virus (AAV) vectors [48-50]. Immunomodulation within the gene therapy realm extends to managing immune responses directed toward both the transgene product and the viral vector. Innovative techniques, such as B cell depletion, rapamycin nanoparticles, and therapeutic regimens featuring rituximab and sirolimus, have emerged as promising avenues for attenuating immune reactions and augmenting outcomes in gene therapy endeavors [49,50]. Furthermore, the deployment of immunomodulatory agents in gene therapy mirrors the paradigm of immune response management in organ transplantation, underscoring the imperative of comprehending and regulating immune reactions to engender successful gene therapy outcomes [49]. Combining immunomodulation strategies into gene therapy protocols significantly heightens gene therapy modalities' safety, efficacy, and durability. By judiciously modulating immune responses, researchers endeavor to optimize the therapeutic efficacy of gene therapy, while mitigating adverse reactions and fostering superior patient outcomes.

Nanotechnology Applications

Based on the provided search results, it seems that nanotechnology applications have not been directly addressed in the context of surgical management of SPS. While nanotechnology holds promise in medicine, including targeted drug delivery and tissue engineering, its specific application in treating PSD must be discussed in the retrieved information. The results emphasize various surgical techniques, emerging minimally invasive approaches like EPSiT, and the importance of individualized treatment plans based on patient factors and surgeon expertise [3,4,25]. While other emerging approaches, such as laser hair removal and regenerative therapies like platelet-rich plasma, are mentioned, nanotechnology is not explicitly mentioned [3,51].

AI in Surgical Planning

AI is profoundly impacting surgical planning, revolutionizing preoperative preparation, intraoperative guidance, and the realm of surgical robotics. In preoperative planning, AI is a valuable tool for surgeons, facilitating the analysis of extensive medical records, and imaging data to optimize surgical interventions. Notably, deep learning algorithms, a subset of AI, spearhead advancements in preoperative planning by enabling anatomical classification, detection, segmentation, and image registration. This breakthrough technology enhances accuracy and efficiency in identifying abnormalities, guiding emergency care and surgical decision-making with unprecedented precision [52]. Moreover, AI assumes a pivotal role in intraoperative guidance, particularly in the domain of minimally invasive surgery (MIS). The cornerstone of MIS, computer-assisted intraoperative guidance, stands to benefit significantly from AI's sophisticated learning strategies. AI's contribution to tissue tracking is particularly significant, as it is a critical component of surgical navigation. Accurate tracking of tissue deformation is paramount for effectively guiding surgical procedures. AI algorithms are pivotal in advancing this facet of surgical navigation, augmenting surgical precision and patient outcomes [53]. In addition, AI-driven surgical robotics are reshaping the landscape of surgical procedures by providing invaluable assistance to surgeons in instrument manipulation and positioning. These robotic systems, propelled by AI technology, empower surgeons to concentrate on the intricate aspects of surgery while mitigating fluctuations during procedures. By enhancing surgical skills and optimizing workflow, AI-driven surgical robotics strive to improve surgical interventions' precision, safety, and efficiency. Ultimately, the integration of AI in surgical robotics holds immense promise for optimizing patient care and elevating the standards of surgical practice [54].

## Conclusions

In conclusion, this review underscores the significant strides made in the surgical management of SPS. Tracing the evolution from traditional techniques to modern innovations, such as laser therapy and robotic-assisted surgery, has illuminated a dynamic landscape of treatment options. Moreover, insights from outcomes research have provided invaluable guidance for clinical decision-making, particularly regarding postoperative pain management, recurrence rates, and patient satisfaction. Moving forward, the implications for clinical practice are clear: surgeons and healthcare providers must remain vigilant in adopting and adapting to these advancements to optimize patient care. A personalized approach, tailored to individual patient needs and circumstances, is paramount. However, there remains ample room for further research. Longitudinal studies comparing the efficacy and safety of different surgical techniques, coupled with investigations into minimally invasive approaches and biomaterials for wound healing, hold promise for improving outcomes and refining treatment protocols.
